# A Role for T-Lymphocytes in Human Breast Cancer and in Canine Mammary Tumors

**DOI:** 10.1155/2014/130894

**Published:** 2014-02-02

**Authors:** Maria Isabel Carvalho, Isabel Pires, Justina Prada, Felisbina L. Queiroga

**Affiliations:** ^1^CECAV, Department of Veterinary Sciences, University of Trás-os-Montes and Alto Douro, 5001-801 Vila Real, Portugal; ^2^Department of Veterinary Sciences, University of Trás-os-Montes and Alto Douro, 5001-801 Vila Real, Portugal; ^3^Center for the Study of Animal Sciences (CECA), University of Porto, Porto, 4480 Vila do Conde, Portugal; ^4^Center for Research and Technology of Agro-Environment and Biological Sciences (CITAB), University of Trás-os-Montes and Alto Douro, 5001-801 Vila Real, Portugal

## Abstract

Chronic inflammation in the tumor microenvironment has a prominent role in carcinogenesis and benefits the proliferation and survival of malignant cells, promoting angiogenesis and metastasis. Mammary tumors are frequently infiltrated by a heterogeneous population of immune cells where T-lymphocytes have a great importance. Interestingly, similar inflammatory cell infiltrates, cytokine and chemokine expression in humans and canine mammary tumors were recently described. However, in both species, despite all the scientific evidences that appoint for a significant role of T-lymphocytes, a definitive conclusion concerning the effectiveness of T-cell dependent immune mechanisms has not been achieved yet. In the present review, we describe similarities between human breast cancer and canine mammary tumors regarding tumor T-lymphocyte infiltration, such as relationship of TILs and mammary tumors malignancy, association of ratio CD4+/ CD8+ T-cells with low survival rates, promotion of tumor progression by Th2 cells actions, and association of great amounts of Treg cells with poor prognostic factors. This apparent parallelism together with the fact that dogs develop spontaneous tumors in the context of a natural immune system highlight the dog as a possible useful biological model for studies in human breast cancer immunology.

## 1. Introduction

The mammalian immune system comprises a coordinated and finely controlled series of interactions involving cells and molecules and has an essential role on species survival and adaptation all over the years [1]. The immune system has the important task of distinguishing between “self” and “nonself,” providing protection against foreign pathogens, maintaining at the same time tolerance to self-antigens [2, 3].

Cancer is a progressive process that arises from a well-defined step where somatic cells acquire activating (oncogenes) or deactivating (tumor suppressor genes) mutations [4, 5]. All types of cancer are caused by the progressive and uncontrolled growth of transformed cells and the control of this disease requires the ablation and destruction of all the malignant cells without damaging the patient. To attain this assignment the own body has to distinguish between the cells of the tumor and other cellular counterparts [3, 6]. However, unfortunately, many tumors continue to grow progressively and expand, which demonstrates that immune system is not always effective and fails on its protective role against tumor development [3, 7].

Decades of intensive investigation left clear that the interplay between immunity and cancer is complex [8]. One example of this high complexity is the phenomena of “*Cancer Immunoediting*.” Cancer cells constantly modulate the host antitumor immune response in a process called *immunoediting*. During this process, the balance between antitumor and tumor-promoting immunity can be tilted to protect against the neoplasia development or, on the contrary, to support tumor growth. Immunoediting is characterized as a three-phase process including *elimination phase* (immunosurveillance), *equilibrium phase*, and *escape phase* [5]. Therefore, the immune system can release factors that promote neoplastic cells survivor, growth, and invasion. Thus, paradoxically, immune system acts as an extrinsic tumor-suppressor but can also promote cancer initiation, promotion, and progression [9].

## 2. Chronic Inflammation and Cancer

The role of chronic inflammation in cancer was first proposed by Rudolf Virchow in 1863, when he observed the presence of leucocytes in neoplastic tissues. Virchow postulated that an inflammatory milieu promotes a cellular environment that drives the initiation and development of carcinogenesis (reviewed in [10, 11]).

Inflammatory responses play a crucial role at different stages of tumor development [12, 13]. Innate immune cells that infiltrate tumors participate in an extensive and dynamic crosstalk with cancer cells and some of the molecular events that mediate this dialog have been revealed [6, 14]. The most relevant molecular mechanisms include increased production of proinflammatory mediators, such as cytokines, chemokines, reactive oxygen intermediates, increased expression of oncogenes, COX-2 (cyclooxygenase-2), 5-LOX (5-lipoxygenase), and MMPs (matrix metalloproteinases), and proinflammatory transcription factors such as NF-*κ*B (nuclear factor *κ*B), STAT3 (signal transducer and activator of transcription 3), AP-1 (activator protein 1), and HIF-1*α* (hypoxia-inducible factor 1*α*). These proinflammatory mediators potentiate tumor cell proliferation, transformation, metastasis, invasion, angiogenesis, chemoresistance, and radioresistance [11, 15–17].

So, *why does inflammation potentiate cancer development rather than protect against it?* In fact, chronic inflammation is considered important in the promotion of cellular proliferation and cancer progression by enhancing angiogenesis and tissue invasion [5, 13], releasing products that promote carcinogenesis in nearby cells and accelerating genetic mutations through a state of malignancy [7]. Finally, through cancer-derived products, immune and regulatory cells are recruited and the weak tumor antigenicity subverts immune cells in order to support cancer progression [5, 18].

## 3. Adaptive Immunity and Cancer Development: A Role for T-Lymphocytes 

In neoplastic lesions, the role of infiltrating T-lymphocytes is often paradoxical. Despite the evidence that the responses of T-lymphocyte can destroy tumor cells “in situ,” these responses appear to be frequently ineffective in the elimination of the established cancer [19, 20]. In fact, patients with cancer present a deficient immune response to tumor antigens. However, this deficient immune response is clearly different from immunosuppression observed in patients receiving high doses of corticosteroids and/or chemotherapy. The term “immune dysfunction” seems the most appropriate to describe the changes observed. The mechanisms that support this “immune disorder” include *barriers that prevent recognition of the tumor by immune cells and also lymphocyte dysfunction* [21].

The *barriers that prevent recognition of the tumor* by immune cells include several mechanisms such as sequestration of tumor associated antigens and major histocompatibility complex (MHC) molecules, loss of costimulatory molecules and other molecules required by cytotoxic T-cells. These mechanisms represent a barrier for the total elimination of tumor [19, 22, 23]. In respect to the *lymphocyte dysfunction* that seems to be present in cancer patients, a tumor-directed immune response involving cytotoxic CD8+ T-cells, T helper 1 (Th1) cells, and natural killer (NK) cells appears to protect against tumor development and progression. Contrarily, the immune responses that involves B-cells, the activation of chronic humoral immunity and/or a T helper 2 (Th2) polarized response and polarized innate inflammatory cells in the tumor, can promote tumor development and progression. This balance between a protective cytotoxic response and a harmful humoral or Th2 response can be regulated systemically by the general immune status of the individual [20, 24].

In this context, the question that arises is *what is the reason why the responses mediated by CD8+ cytotoxic T-lymphocytes are not effective in eradicating the tumor and how can the CD4+ T-cells be involved in neoplastic progression of this disease?*

A part of the response has already been described above and is related to tumor escape mechanisms from cytotoxic CD8+ T-cells action. Another important mechanism appears to be related to the polarity of the responses of CD4+ T-cells in relation to the primary site of cancer and/or their distant metastases [24] and the imbalance of the normal ratio of Th1/Th2 cells [25].

CD4+ T-cells are activated in response to soluble factors and can be classified into categories, Th1 and Th2. After stimulation, the Th1 cells secrete interferon gamma (IFNg), transforming growth factor beta (TGF*β*), tumor necrosis factor alpha (TNF), and interleukin 2 (IL-2). These cytokines cooperate with the functions of cytotoxic CD8+ T-cells, producing a tumoricidal activity. In contrast, Th2 cells express interleukin (IL) 4, 5, 6, 10, and 13 that induce anergy of T-cells and loss of cytotoxicity, while increasing the humoral immunity (lymphocyte B function). Thus, Th1 cell responses benefit antitumor immunity, whereas Th2 cell responses produce a down-regulation of antitumor cell mediated immunity and increase the humoral protumourigenic responses [24, 26, 27].

Although the immune dysfunction in patients with cancer is now better understood with the perception of Th1 and Th2 regulation, what is responsible for this dysfunction remains to be determined. One possibility is that the number of Th1 cells or their precursors are reduced, decreasing one line of defense against cancer progression and metastasis. Another possibility is the important role played by *regulatory T-cells* and *immature myeloid cells* in the antitumor immune suppression observed in patients with breast cancer and other type of neoplasms [25, 28].

*Regulatory T-cells* (Treg cells) are a distinct group of lymphocytes with immunosuppressive properties that usually maintain immune tolerance [29]. Treg cell suppressive activity is beneficial by restricting T cell response against self-antigens and preventing inflammatory and autoimmune diseases. In cancer, their inhibitory role in limiting immune response against “pseudo-self antigens” from tumor origin avoids an effective antitumoral immune response and often culminates into negative outcomes for the patient. These cells may play an important deleterious role in cancer immunopathology due to their potent suppressive activity of both T-cell activation and effector functions [20, 30, 31].

*Immature myeloid cells* express MHC class I molecules suggesting that they can induce cytotoxic T-cells anergy by binding to T-cell receptor (TCR) complex in absence of costimulatory signals [32, 33].

In the last years, a new subset of CD4+ (helper) T-cells, termed *Th17 cells*, has been characterized. Th17 subset secretes IL-17, IL-21, and IL-22 and plays critical roles in the pathogenesis of inflammatory and autoimmune diseases, as well as in host protection against pathogens. Although some data suggest the importance of Th17 cells for tumor immunity, conclusions regarding the functional role of Th17 cells remain controversial [34–36]. Even though some studies indicate that mouse Th17 cells support a positive anti-cancer immunity, the Th17 cells with intratumoral location are probably responsible for chronic tissue inflammation and appear to have a tumor-promoting effect [35, 37, 38].

## 4. T-Lymphocytes and Human Breast Cancer: Friends or Foes?

In humans, the study of the inflammatory infiltrate, mainly T-lymphocytes, has been subject of great interest associated not only with breast cancer [19, 23, 39], but also with other types of neoplasias, including seminoma [40, 41], melanoma [42, 43], colorectal [44, 45], cervical [46], ovarian [47, 48], urothelial [49], and gastric cancer [50].

In breast cancer, an important role has been attributed to inflammatory cells, as well as cytokines produced by them. A large number of observations suggest that inflammatory cells are not “innocent spectators,” but, contrarily, they might conspire with the tumor cells favoring tumor development and progression [8]. However, the prognostic significance of infiltrating T-lymphocytes is still subject to considerable debate [24, 51], because no definitive conclusions have been reached so far. The T-lymphocytes infiltrate appear, according to some researchers, associated with a better prognosis, whereas in other cases is related to a decline in overall survival.  Table 1 illustrates some of the most relevant studies in this area in the last two decades.

More recently, investigations that focus on understanding the functions of Treg cells and Th17 cells in mammary carcinogenesis have been published; however, the results of the various studies are also quite controversial [69–71].

Lee and collaborators [69] investigated, by immunohistochemistry, whether the presence of FOXP3-positive Treg cells was associated with prognostic factors, such as stage or histologic grade. FOXP3-positive Treg cells were, in this study, an independent prognostic factor for overall survival and progression free survival. The improved survival times were associated with highly infiltrating FOXP3-positive Treg cells.

Another study, on the contrary, showed that the increased number of Foxp3 Treg cells was significantly correlated with lymph node metastasis and immunopositivity for Ki-67, which indicates a probable relationship with a worse prognosis. [70].

Wang and colleagues assessed the Th17 and Treg cells by flow cytometry and observed that Th17 and Treg cells accumulation in the tumor microenvironment of breast cancer occurred in early stages of disease. With tumor progression, Th17 cell infiltration gradually decreased and there was accumulation of Treg cells [71]. So, this last study indicates that an increase in the number of Treg cells is associated with tumor progression.

The apparent controversy among distinct studies emphasizes the need for further research on this topic. A clear understanding about the role of T-lymphocytes in breast cancer is essential for the development of new therapeutic strategies in a near future.

## 5. T-Lymphocyte Infiltrate in Canine Mammary Tumors

Canine mammary tumors are a spontaneous neoplasia that occurs frequently in the clinical practice [72, 73]. Despite the high number of studies published on this subject in the last decades, little is known about the role of tumor inflammatory infiltrate in cancer development and/or progression. In dogs, the first studies focused on inflammation and cancer have been performed in other type of tumors including transmissible venereal sarcoma [74], benign oral papilloma [75], cutaneous histiocytoma [76], and seminomas [77]. Studies investigating the role of inflammation in canine mammary tumors were only recently published (Table 2) [78–83].

In canine transmissible venereal sarcoma, the quantity of T-lymphocytes is higher in the group of tumors that exhibit spontaneous regression or stable growth, comparatively with the tumors that exhibit a progressive growth [74]. In canine oral papilloma, similar to humans [84], the maximum number of T-cells that infiltrate the tumor occurs during rapid tumor regression [75]. In canine cutaneous histiocytoma, in the same way, a lymphocytic infiltrate represents the morphological expression of one antitumor immune response, which correlates with observations of spontaneous regression “in vivo” [76, 85]. In turn, in canine seminomas [77], in accordance with what occurs in human seminomas [40, 41], infiltrating lymphoid cells consist mainly in T-lymphocytes, especially CD8+ cells, which means that the reaction of the body against neoplastic cells is mainly cytotoxic. This, together with the number of MHC I positive cells and a high amount of antigen presenting cells observed, suggests, according to the authors, that inflammatory cells exhibit a role in antitumor response [77]. This might explain the biological behavior of these tumors that rarely metastasize and the favorable prognosis that often presents. Interestingly, in 2007, Horiuchi and collaborators [86] refer that in animals with cancer, a smaller amount of Th1 cells and a significant larger amount of Th2 cells, compared to healthy ones, was observed. Considering that Th2 cells have an action that promotes tumor progression, these results have come refute what is already known in human works and relaunch the interest in this subject in dogs.

In canine mammary tumors, as previously stated, there are only a very limited number of studies, all of them recently published, that focus on effect of T-lymphocytes infiltrate and malignancy [78–83]. In malignant mammary tumors, abundant lymphocyte infiltrates are frequently found, but the characteristics associated with lymphocyte infiltration in these tumors remain largely unknown. As in humans, the controversy among distinct reports remains an important issue to be clarified (Table 2).

According to Estrela-Lima and collaborators [78], lymphocytes represent the predominant cell type in the tumor infiltrate. The relative percentage of CD4+ T-cells was significantly greater in metastasized tumors, while the percentage of CD8+ T-cells was higher in cases without metastasis. Consequently, the CD4+/CD8+ ratio was significantly increased in cases with metastasis and was associated to lower survival rates. Authors defend that the intensity of lymphocytic infiltrate and the CD4+/CD8+ ratio may represent important survival prognostic biomarkers for canine mammary carcinomas.

In one study performed by Kim and colleagues [79], immunohistochemistry, immunoblotting, and reverse transcriptase-polymerase chain reaction were used to evaluate tumor infiltrating lymphocytes (TILs) and the presence of related cytokines, as well as the expression of breast cancer susceptibility gene-1 (BRCA1). The results of this study revealed a correlation between expression of interleukin (IL)-1 and IL-6 and tumor metastasis. An association among the expression of TILs, cytokines, and mutation of BRCA1 was also verified, suggesting that all of these factors may play a role in tumor progression.

In another study developed by our team [80], CD3+ T-lymphocytes were evaluated in three distinct areas: within the tumor, in the periphery of the tumor and in the adnexal non-tumoral mammary gland. We observed a tendency towards an association of a higher number of CD3+ tumor infiltrating T-lymphocytes and a shorter overall survival. However, interestingly, only for CD3+ T-lymphocytes in the adnexal non-tumoral mammary gland, a statistically significant relationship was observed, with a higher number of lymphocytes conferring a reduced overall survival. This could indicate that CD3+ T-lymphocytes in adnexal non-tumoral mammary gland were implicated in tumor progression and survival, showing that its protumourigenic immune responses may somehow be the starting point for the growth and progression of tumor cells.

Saeki and collaborators [81] accessed the number of tumor infiltrating T-lymphocytes, B-lymphocytes, and antigen presenting cells by immunohistochemistry. As a result, the authors found a statistically significant increase in the number of intratumoral T-lymphocytes in malignant tumors compared with benign ones. The results of this study indicate a positive relationship between a high number of TILs and increased canine mammary tumors malignancy.

Very recently, Kim and colleagues [83] demonstrated, by immunohistochemistry, that the degree of lymphocyte infiltration was significantly higher in canine mammary carcinomas with lymphatic invasion and high histologic grade, suggesting the importance of lymphocytes on tumor aggressiveness and greater malignant behavior.

Treg cells, whose activity is closely associated with the transcription factor FOXP3, have a suppressive action on T-lymphocytes antitumor responses [87, 88]. In dog, there are few studies that focus on the action of Treg cells in tumor development and progression [82, 89–91]. Dogs with cancer had increased numbers of Treg cells in their peripheral blood and tumor-draining lymph nodes compared to healthy animals [89].

In dog mammary tumors, a recent study by Kim and Colleagues [82] described abundant Treg cells associated with high histological grade and lymphatic invasion. The number of Treg cells infiltrating intratumoral areas was markedly increased in tumors with poor prognostic factors, such as high histological grade of malignancy, presence of lymphatic invasion, and presence of tumoral necrosis. These findings suggest that Treg cells might play a key role in canine mammary tumors progression. Furthermore, the amount of intratumoral Treg cells may provide a new prognostic factor when assessing survival times, which may in turn lead to the development of new immunologic therapies.

**Figure 1:**
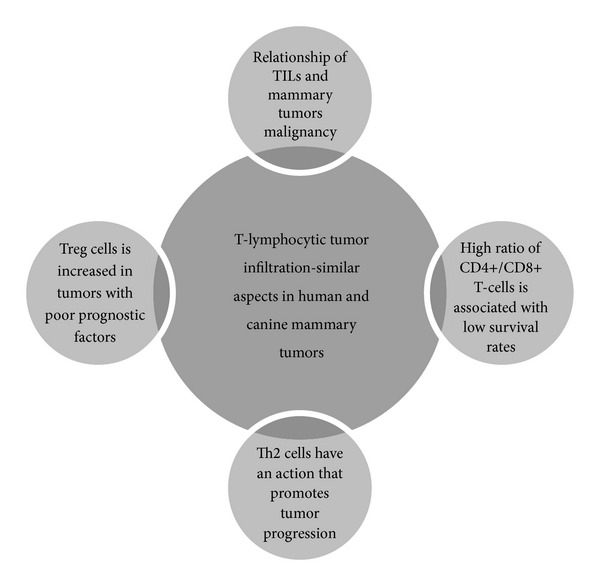
Similarities between human breast cancer and canine mammary tumors regarding tumor T-lymphocyte infiltration.

The studies described above, concerning canine mammary tumors, describe results that are in agreement with those from studies already published in human breast cancer which may be an indication of similar cancer immunologic aspects between the two species (Figure 1).

## 6. Final Remarks

The dog has been proposed, by various authors and throughout several decades, as a model for the study of spontaneous malignancies in humans. This hypothesis is supported by the knowledge that development of spontaneous tumors in dogs and humans is a phenomenon highly incident, sharing many features: histological appearance, tumor genetics, molecular targets, biological behavior, and response to conventional therapy [92–94]. The recent sequencing of the canine genome and the evidence of its similarity to the human counterpart emphasized even more the dog as an attractive model for cancer research [73, 95, 96].

Breast cancer remains a major clinical challenge with considerable mortality both in humans and dogs [72, 97]. Scientific evidences support that, in both species, alterations of inflammatory components within the tumoral microenvironment have a significant role during important steps of carcinogenesis. Additionally, dogs develop spontaneous tumors in the context of a natural immune system [94] which make them an attractive and viable target for immune therapeutic modulation [5, 8, 80, 97, 98].

In the present review, we describe similarities between human breast cancer and canine mammary tumors regarding tumor T-lymphocyte infiltration, such as relationship of TILs and mammary tumors malignancy, association of ratio CD4+/CD8+ T-cells with low survival rates, promotion of tumor progression by Th2 cells actions, and association of great amounts of Treg cells with poor prognostic factors.

We believe that the current state of knowledge could be the basis for a broader and deeper discussion concerning the role of inflammation in dog tumors, especially in canine mammary cancer. Nevertheless, it remains to be clarified the role of the inflammatory infiltrate in tumors of high biological aggressiveness and thus elucidate the T-cell subtypes implicated in the progression of these neoplasms. The identification of specific subtypes and the clarification of the involved pathways, may serve as a basis for the establishment of new therapeutic strategies. In this sense, the development of an active immunization throughout the design of new anticancer-vaccines is expected both in humans and dogs.

In human breast cancer, it was already postulated that vaccination could induce an expansion of CD8+ cytotoxic T-lymphocytes capable of rejecting tumor cells via recognition of tumor-associated antigenic epitopes, located on the surface of cancer cells [99, 100]. The development of anti-cancer vaccines may lead to the establishment of immunological memory, thereby preventing tumor recurrence with potential advantages in inducing antitumor immune responses in both species [11]. Dogs with mammary cancer develop metastatic disease in a shorter time compared with the humans, due to their smaller longevity which make them particularly good models for study the metastatic process and thus testing new therapeutic modalities [73, 94]. The similarities pointed out in this review support the use of dog with mammary cancer as a reliable biological model to study human breast cancer immunology, providing an attractive opportunity for therapeutic clinical studies in the scope of comparative oncology.

## Figures and Tables

**Table 1:** Studies of a generalized lymphocytic infiltration in human breast cancer.

Author	Year	Patients (*n*)	Analysis	Location	Type	Comments
Aaltomaa and Lipponen [52]	1992	489	Semiquantitative (none/mild-moderate-severe)	Peritumoral Intratumoral	Lymphocytic infiltration	Better prognosis, positive correlation with the lack of regional lymph nodes involvement, with a smaller diameter of the tumor, with a lower histologic grade and with a larger time free of disease

Carlomagno et al. [53]	1995	1257	Semiquantitative (absent/present)	Peritumoral	Lympho-plasmacytic infiltration	Poorer overall survival in multivariate survival analysis in particularly patients with grade I and II

Ménard et al. [54]	1997	1919	Semiquantitative (absent/present)	Intratumoral	Lymphoid infiltration	Better overall survival in patients <40 years of age, no significant association in patients >40 in multivariate survival analysis

Marsigliante et al. [55]	1999	90	Quantitative (computerised counting)	Intratumoral	T-lymphocyte infiltration	CD3+ TILs was directly correlated to age, lymph node negative patients had tumors infiltrated by fewer CD4+ TILs with respect to lymph node positive patients

Georgiannos et al. [56]	2003	60	Semiquantitative (0 = none; 1 = rare cells; 2 = moderate numbers; and 3 = many positive cells)	Intratumoral	Lymphocytic infiltration	A significant association was found between the intensity of TIL and the number of positive nodes

Campbell et al. [25]	2005	84 patients 26 healthy volunteers	The flow cytometry analysis was performed using CELLQuest software (BD Biosciences)	Peripheral blood and bone marrow aspirates	Peripheral blood lymphocytes	The percentages of both CD4+ and CD8+ cells were significantly lower in patients with breast cancer compared to healthy controls. A correlation was observed between number of micrometastases in the bone marrow and T cell responsiveness

Lee et al. [57]	2006	679	Semiquantitative (none-mild-moderate-severe)	Peritumoral Intratumoral	General inflammation infiltrate	Better recurrence-free survival and overall survival in multivariate survival analysis

Macchetti et al. [19]	2006	23	Flow cytometry quantitative analysis	Intratumoral	Lymphocytic infiltration	In the patients with lymph node metastasis, an increased mean percentage of tumor infiltrating CD4+ T-cells, but not CD8+ T-cells was observed and was correlated with worse prognosis

Al Murri et al. [58]	2008	168	Quantitative analysis	Peritumoral Intratumoral	CD4 and CD8	No significant association

Calabrò et al. [59]	2009	155	Quantitative analysis microarray based screening for Li-associated genes		Lymphocytic infiltration	Poorer overall survival in ER+ patients and better overall survival in ER− patients

Rakha et al. [60]	2009	1597	Semiquantitative (mild-severe)	Peritumoral Intratumoral	General inflammation	Better recurrence-free survival and overall survival

Rody et al. [61]	2009	1263	Quantitative analysis	Peritumoral Intratumoral	CD3	Better recurrence free survival in cases who had HER-2+

Matkowski et al. [62]	2009	88	Semiquantitative (percentage of positive stained cells: 0 = none, 1 = up to 33%, 2 = 33–66%, 3 = more than 66%; intensity of the lymphocytic infiltrate: 1-low, 2-moderate, 3-high)	Intratumoral	Lymphocytic infiltration	In early breast cancer the presence of CD8+ and CD4+ cells was correlated with lymph node involvement and unfavorable prognosis

Baker et al. [63]	2011	1953	Quantitative analysis (TMA)	Peritumoral Intratumoral	CD8	Better cancer-specific survival in only high grade ER− tumor in multivariate survival analysis whereas poorer cancer specific survival in low grade ER+ tumor in univariate analysis

Ladoire et al. [64]	2011	162	Semiquantitative (none-mild-moderate-severe)	Peritumoral Intratumoral	CD8	Better recurrence-free survival after chemotherapy CD8/FOXP3 ratio independent predictive factor associated with improved recurrence-free and overall survival after chemotherapy

Liu et al. [65]	2011	1270	Quantitative analysis	Peritumoral Intratumoral	CD8	No significant association

Mahmoud et al. [66]	2011	1334	Quantitative analysis (TMA)	Peritumoral Intratumoral	CD8	Better cancer-specific survival in multivariate survival analysis. Better recurrence free survival and cancer specific survival in only ER− in univariate survival analysis. Whereas no significant association in ER+

West et al. [67]	2011	255	Quantitative analysis microarray based on information in the BioGPS gene portal	Intratumoral	Lymphocytic infiltration	TIL that express cytotoxic markers was strongly associated with favorable outcome after anthracycline based treatment of ER− breast cancer patients

Ruffell et al. [68]	2012	20	Flow cytometry, immunohistochemistry, and confocal immunofluorescence quantitative analysis	Intratumoral	Leukocyte infiltration	Tumors from breast cancer patients treated with neoadjuvant chemotherapy contained an increased CD8/CD4 T-cell ratio compared with tumors removed from patients treated primarily by surgery alone

**Table 2:** Studies of T-lymphocytic infiltrate in Canine mammary tumors (CMT).

Author	Year	Patients (*n*)	Type	Comments
Estrela-Lima et al. [78]	2010	51	T-lymphocyte infiltration	Animals with high proportions of CD4+ and low CD8+ T-cells had lower survival rates

Kim et al. [79]	2010	58	T-lymphocyte infiltration	Association between the expression of TILs, cytokines, and mutation of BRCA1 suggests that all of these factors may play a role in tumor progression

Carvalho et al. [80]	2011	57	T-lymphocyte infiltration	Tendency for an association of a higher number of CD3+ TILs and a shorter overall survival. CD3+ T-lymphocytes in the adnexal nontumoral mammary gland revealed a statistically significant relationship with overall survival

Saeki et al. [81]	2012	140	Lymphocytic infiltration	Relationship of TILs and canine mammary tumors malignancy

Kim et al. [82]	2012	37	Regulatory T-cells (Treg)	The number of Treg cells is increased in tumors with poor prognostic factors, such as high histological grade, lymphatic invasion, and necrosis

Kim et al. [83]	2013	47	Lymphocytic infiltration	Intense lymphocyte infiltration was associated with aggressive histologic features (higher histologic grade; lymphatic invasion)
